# A second-generation “hypoxia in a pill” rescues neurodegenerative phenotypes across distinct mouse models

**DOI:** 10.1073/pnas.2618654123

**Published:** 2026-10-09

**Authors:** Hong Wang, Eizo Marutani, Luca Zazzeron, Marissa Menard, Laura Volpicelli-Daley, Fumito Ichinose, Vamsi K. Mootha

**Affiliations:** ^a^ HHMI and Department of Molecular Biology, Massachusetts General Hospital, Boston, MA 02114; ^b^Department of Systems Biology, Harvard Medical School, Boston, MA 02115; ^c^ Broad Institute of Massachusetts Institute of Technology and Harvard, Cambridge, MA 02142; ^d^ Anesthesia Center for Critical Care Research of the Department of Anesthesia, Critical Care and Pain Medicine, Massachusetts General Hospital and Harvard Medical School, Boston, MA 02114; ^e^ Killion Center for Neurodegeneration and Experimental Therapeutics, Department of Neurology, University of Alabama at Birmingham, Birmingham, AL 35294

**Keywords:** hypoxia, hemoglobin, HIF-2α, neurodegeneration, hyperoxia

## Abstract

A growing body of preclinical research is demonstrating the therapeutic potential of chronic, continuous hypoxia (11% FIO_2_) for rare and common diseases. However, the chronic delivery of hypoxic gas poses both practical challenges and long-term safety concerns. We previously introduced a small-molecule, “hypoxia-in-a-pill” combining a hemoglobin affinity enhancer (GBT440) to limit oxygen delivery with a HIF-2α inhibitor (PT2399) to prevent detrimental compensatory erythropoiesis. Although this small-molecule regimen extended the lifespan of the *Ndufs4* knockout (KO) mouse model of mitochondrial complex I deficiency and Leigh syndrome, its efficacy did not match chronic 11% FIO_2_. Here we optimize this regimen using GBT601, a second-generation hemoglobin affinity enhancer with longer half-life and greater hemoglobin occupancy, and show it rescues key neurodegenerative phenotypes in multiple models. When we initiate therapy in 50 d old *Ndufs4* KO mice with advanced disease, GBT601 monotherapy alleviated neurological disease phenotypes and extended median lifespan from 62 to 105 d, while dual therapy with the GBT601/PT2399 combination extended median lifespan to 158 d. When initiated after onset of advanced disease in a mouse model of Friedreich’s ataxia, the combination halted further progression of motor phenotypes. In a mouse model of Parkinson’s disease due to α-synuclein toxicity, initiating the GBT601/PT2399 combination after onset of motor dysfunction attenuates brain hyperoxia and lipid peroxidation and reverses motor phenotypes. Importantly, the combination maintained body weight without inducing any signs of pulmonary hypertension. Our findings motivate further preclinical and clinical evaluation of our “hypoxia in a pill” approach for diseases with high unmet need.

## Significance

Chronic, continuous systemic hypoxia is emerging as a promising preclinical therapy for a variety of rare and common diseases, however its clinical implementation is hindered by practical barriers and potential long-term side effects. Building on our prior work, we introduce a second-generation “hypoxia in a pill” drug combination and show that it induces therapeutic hypoxia with high efficacy. This regimen is able to halt and even reverse some of the neurodegeneration phenotypes in mouse models without the detrimental cardiac effects typically associated with chronic low-oxygen environments, thereby offering a pharmacologically tractable form of therapeutic hypoxia.

Although seemingly counterintuitive, a mounting body of research is demonstrating that chronic moderate hypoxia can have widespread therapeutic benefits (1). Epidemiological studies have long suggested the benefits of living at high altitude. For instance, residents of Swiss (2) and Austrian (3) Alps and the western United States (4) exhibit lower mortality from cardiovascular disease, stroke, cancer, and Alzheimer’s disease. Anecdotal evidence suggests that high altitude may ameliorate motor symptoms in Parkinson’s disease (PD) (5). A notable “natural experiment” involving ~20,000 soldiers from the Indian army stationed at high altitude (3,200 to 4,800 m, equivalent to an inspired oxygen of 11 to 13% at sea level) for 3 y revealed a lower incidence of hypertension, type 2 diabetes, and ischemic heart disease compared to that of ~130,000 peers stationed at sea level (6). While many other environmental variables such as climate, solar radiation, diet, and activity confound these human data, preclinical research has supported reduced oxygen as a candidate driver of these benefits.

Preclinical mouse studies beginning in 2016 showed that continuous exposure to 7 to 11% oxygen provides profound protective effects across diverse models, including mitochondrial gray matter disease called Leigh syndrome (7–10), Leigh-like syndrome (11), optic neuropathy (12), Friedreich’s ataxia (FA) (13, 14), multiple sclerosis (15), accelerated aging phenotype (16), recovery from myocardial infarction (17) and ischemic stroke (18, 19), and PD (20). The therapeutic benefits of hypoxia for rare and common forms of neurodegenerative diseases appear to be evolutionarily conserved from mice to *C. elegans* (10, 13, 20). Given the widespread role of molecular oxygen in metabolism and signaling, the therapeutic benefits of hypoxia are likely to be pleiotropic in origin, but key specific mechanisms are being identified. For example, we documented that mouse models of primary or secondary mitochondrial complex I deficiency exhibit brain hyperoxia, which we have shown can be attenuated by breathing chronic, continuous hypoxia (9, 20). Low ambient oxygen also helps to preserve the activity of mitochondrial complex I (10, 13), helps to restore iron-sulfur cluster levels (13), and reduces brain lipid oxygenation (20). In most of these models, traditional antioxidants are not effective and cannot provide a simple substitute for chronic hypoxia.

Harnessing these benefits requires continuous rather than intermittent regimens of hypoxia. In the *Ndufs4* KO model of Leigh syndrome, daily 10-h hypoxic bouts failed to provide benefit (8) while in the *shFxn* model of Friedreich’s ataxia, a 16-h daily regimen even proved detrimental (14). We hypothesized that this failure likely stems from the elevated hematocrit and hemoglobin (Hb) induced by the hypoxic periods, which, upon return to normoxia, creates a state of excess oxygen that is detrimental. In support of this thesis, we showed that pharmacologically inhibiting HIF-2α, which blunts the erythropoietic response to low oxygen, can attenuate these detrimental effects (14).

Safely delivering chronic continuous hypoxia raises practical challenges. The sporting industry has created a variety of portable nitrogen generators, tents, and face masks, that allow athletes to train in environments simulating high altitude. We previously “repurposed” such equipment in a study of healthy volunteers to achieve normobaric hypoxia over 5 d that nadired at 11% oxygen that was safe and well-tolerated (21). It is conceivable that this equipment could be repurposed for long-term hypoxia therapy, though this would be cumbersome.

Chronic exposure to continuous hypoxia is also associated with a number of long-term, detrimental side effects, including pulmonary hypertension (PH) and right ventricular hypertrophy (RVH) in humans (22, 23) and in mice (24). These risks are particularly pronounced during development where neonatal hypoxia interferes with lung airway and vascular development, with reduced long-term survival due to severe PH and RV failure (24, 25). Furthermore, long-term hypoxia is associated with development of paragangliomas and glomus tumors (26). Many of the detrimental side effects of chronic hypoxia are due to prolonged and sustained activation of HIF-2α (27).

These challenges underscore the need to be able to deliver hypoxia in a way that is both practical and safe, motivating us to introduce “hypoxia in a pill,” the first small molecule cocktail for inducing systemic, therapeutic hypoxia (28). Our small molecule approach was based on dual targeting of hemoglobin and HIF-2α using two classes of FDA approved drugs, respectively. First, we used the hemoglobin affinity enhancer GBT440 to decrease oxygen offloading and thereby decrease tissue oxygen levels. Linus Pauling showed in the 1940s that oxygenated hemoglobin—corresponding to a conformation called the “R” state—has a lesser tendency to sickle (29). Small molecule hemoglobin affinity enhancers that stabilize this oxygen bound R conformation have been developed to treat sickle cell anemia (30, 31). However, the decrease in oxygen offloading achieved with GBT440 can in principle induce a compensatory, HIF-2α-dependent erythropoietic response which serves to increase tissue oxygen delivery and therefore limits the effectiveness of GBT440 monotherapy. Hence, we combined GBT440 with the HIF-2α inhibitor PT2399, a class of drugs that has been approved for vHL null renal tumors (28). We showed that treatment with the combination could extend the lifespan of the *Ndufs4* KO mouse model of Leigh syndrome and delay onset of neurological disease (28). A subsequent study showed that daily administration of a structural analog of GBT440 from the same initial chemical series could also extend lifespan in the *Ndufs4* KO mouse model (32), though it is an irreversible covalent binder administered at extremely high doses and never advanced into humans.

While our initial combination consisting of two FDA approved classes (28) provided proof-of-concept, it remained less effective than inhaled 11% oxygen, likely due to the short half-life and limited Hb occupancy by GBT440. Here, we sought to optimize the regimen using GBT601 (Osivelotor), a potent, second-generation, clinical-stage molecule (33–35). GBT601 exhibits greater hemoglobin occupancy and a longer half-life in rats compared with GBT440 (33). In a murine model of sickle cell disease, GBT601 treatment resulted in both an increase in hemoglobin oxygen affinity and a reduction in sickling of red blood cells (RBCs) that exceeded the effects of GBT440 preclinically (36). These pharmacological improvements enable equivalent HbS occupancy at a lower dose, reducing treatment burden. Importantly, GBT601 has already completed safety studies in healthy humans (34).

Here we sought to explore the therapeutic potential of GBT601 alone or the GBT601/PT2399 combination in preclinical mouse models of mitochondrial and neurodegenerative diseases in which inhalational hypoxia has previously been shown to be effective. We show that this combination is tolerated without apparent adverse effects in wild-type (WT) mice, achieves durable systemic and brain hypoxia, and when initiated in advanced stages of disease in these models, is able to alleviate neurodegenerative phenotypes. Importantly, dual targeting of hemoglobin and HIF-2α achieves these results without the detrimental cardiac side effects typically associated with chronic hypoxia.

## Results

### Dual Targeting of Hemoglobin and HIF-2α with GBT601/PT2399 Induces Systemic Hypoxia.

**Fig. 1.**
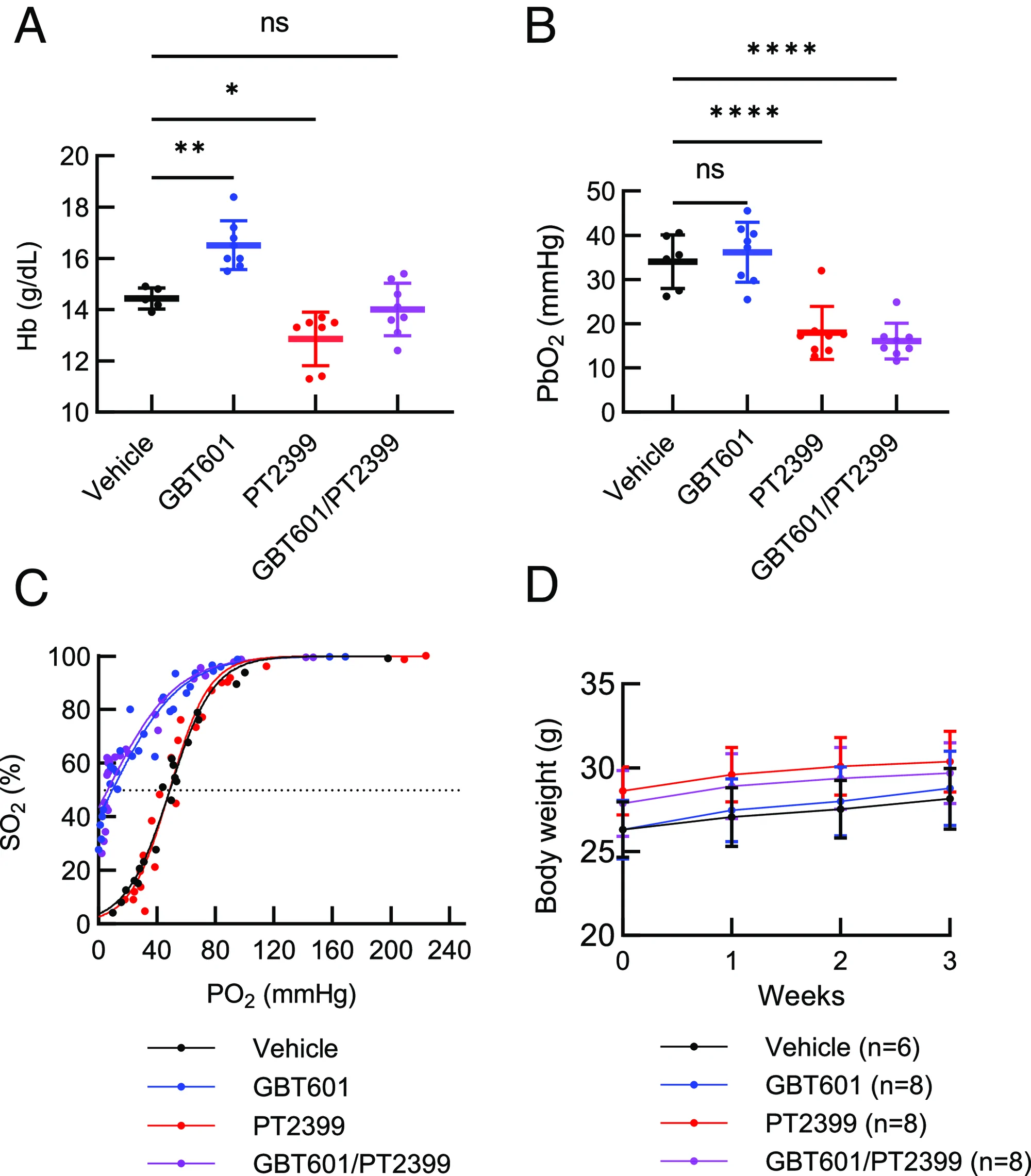
GBT601/PT2399 combination induces systemic hypoxia. (*A*) Hb and (*B*) brain PbO_2_ measurements in vestibular nucleus of 10-wk-old C57BL/6J male mice treated with vehicle, GBT601, PT2399, or the combination for 3 wk (n = 5 to 8 per group). (*C*) oxygen–hemoglobin dissociation curve measurements of 12-wk-old C57BL/6J male mice treated with vehicle, GBT601, PT2399, or the combination for 7 d (n = 4 to 5 per group). (*D*) Body weight of C57BL/6J mice treated with the indicated drugs. Bar plots show the mean ± SD. *P < 0.05, **P < 0.01, ***P < 0.001, ****P < 0.0001; one-way ANOVA with Dunnett’s test for multiple comparisons with vehicle. oxygen–hemoglobin dissociation curve compared by nonlinear regression (extra sum-of-squares F test).

We first measured effects of daily administration of GBT601 and PT2399 on hemoglobin levels and brain tissue oxygen partial pressure (PbO_2_) in WT C57BL/6J mice. We began by treating WT mice with each drug, individually or in combination, by oral gavage daily using a fixed dose previously reported (28, 36). After 3 wk of daily treatment, the average hemoglobin in vehicle-treated mice (n = 5) was 14.4 ± 0.4 g/dL, rose in mice treated with GBT601 alone to 16.5 ± 1.0 g/dL (n = 8, *P* = 0.002) and decreased in those treated with PT2399 alone to 12.9 ± 1.0 g/dL (n = 7, *P* = 0.02). The combination, however, led to Hb levels comparable to those with vehicle treatment (14.0 ± 1.0 g/dL; n = 8, *P* = 0.8). These findings confirm that the erythroid response to GBT601 is blunted by the HIF-2α inhibitor PT2399 (Fig. 1*A*).

To determine whether the drugs were inducing systemic and tissue hypoxia, we made brain PO_2_ (PbO_2_) measurements. Using an optical oxygen probe, we found that mice treated with GBT601 alone had similar PbO_2_ in the vestibular nucleus to the vehicle-treated (34.1 ± 6.1 mmHg vs. 36.2 ± 6.8 mmHg, *P* = 0.8), while those treated with PT2399 had significantly decreased PbO2 17.9 ± 6.0 mmHg) (*P* < 0.0001 vs. vehicle). The combination of GBT601 and PT2399 decreased PbO2 to 16.1 ± 4.0 mmHg (*P* < 0.0001 vs. vehicle) (Fig. 1*B*).

Oxygen dissociation curves demonstrated distinct shifts among treatment groups. Vehicle-treated mice exhibited a typical sigmoidal oxyhemoglobin saturation curve with a P_50_ of 48.9 mmHg (95% CI: 47.1 to 50.7). PT2399 treatment alone did not substantially alter hemoglobin–oxygen affinity, with a P_50_ of 48.7 mmHg (95% CI: 45.7 to 51.9). In contrast, and as expected, GBT601 treatment produced a marked left shift of the oxygen dissociation curve. The P_50_ was significantly reduced to 9.6 mmHg (95% CI: 5.8 to 12.9), reflecting increased hemoglobin–oxygen affinity and enhanced saturation at lower PO_2_ levels. The combination of PT2399 and GBT601 also demonstrated a pronounced leftward displacement, with a P_50_ of 6.4 mmHg (95% CI: 1.8 to 10.1), indicating preserved GBT601-mediated increases in oxygen affinity in the presence of PT2399 (Fig. 1*C*).

Comparable to what we previously observed in mice treated with GBT440 and PT2399, the trajectory of body weight was the same for mice treated with vehicle, GBT601 alone, PT2399 alone and the combination of GBT601/PT2399. (Fig. 1*D*). Thus, the combination of GBT601/PT2399 induces sustained brain tissue hypoxia and is well tolerated in WT mice for at least 3 wk of daily treatment.

### GBT601 Alone or in Combination with PT2399 Alleviates Neurodegenerative Phenotypes and Extends Lifespan in a Mouse Model of Leigh Syndrome.

Leigh syndrome is the most common pediatric manifestation of mitochondrial disease and is characterized by a subacute degeneration of deep gray matter regions. Patients tend to be born developmentally intact and the disease presents as a neurometabolic crisis in infancy or in childhood with characteristic brain MRI changes. To date, more than 110 different disease genes, nearly all of which encode mitochondrial proteins, have been identified (37), and unfortunately, there are no FDA approved medicines. We previously showed that chronic, continuous exposure to 11% FIO_2_ initiated after weaning prevents the formation of brain pathology in the *Ndufs4* KO mouse model of Leigh syndrome due to complex I deficiency (7). As the brain disease is the cause of death in these mice, hypoxic breathing leads to a striking extension in lifespan. We later showed that when initiated after onset of advanced disease, 11% FIO_2_ could halt further neurodegeneration and even reverse MRI lesions and motor phenotypes (8). Moreover, we reported that the first generation “hypoxia in a pill” (28) consisting of the GBT440/PT2399 combination when initiated at the time of weaning could delay onset of disease and prolong median lifespan by 30%, whereas neither drug was effective in isolation.

**Fig. 2.**
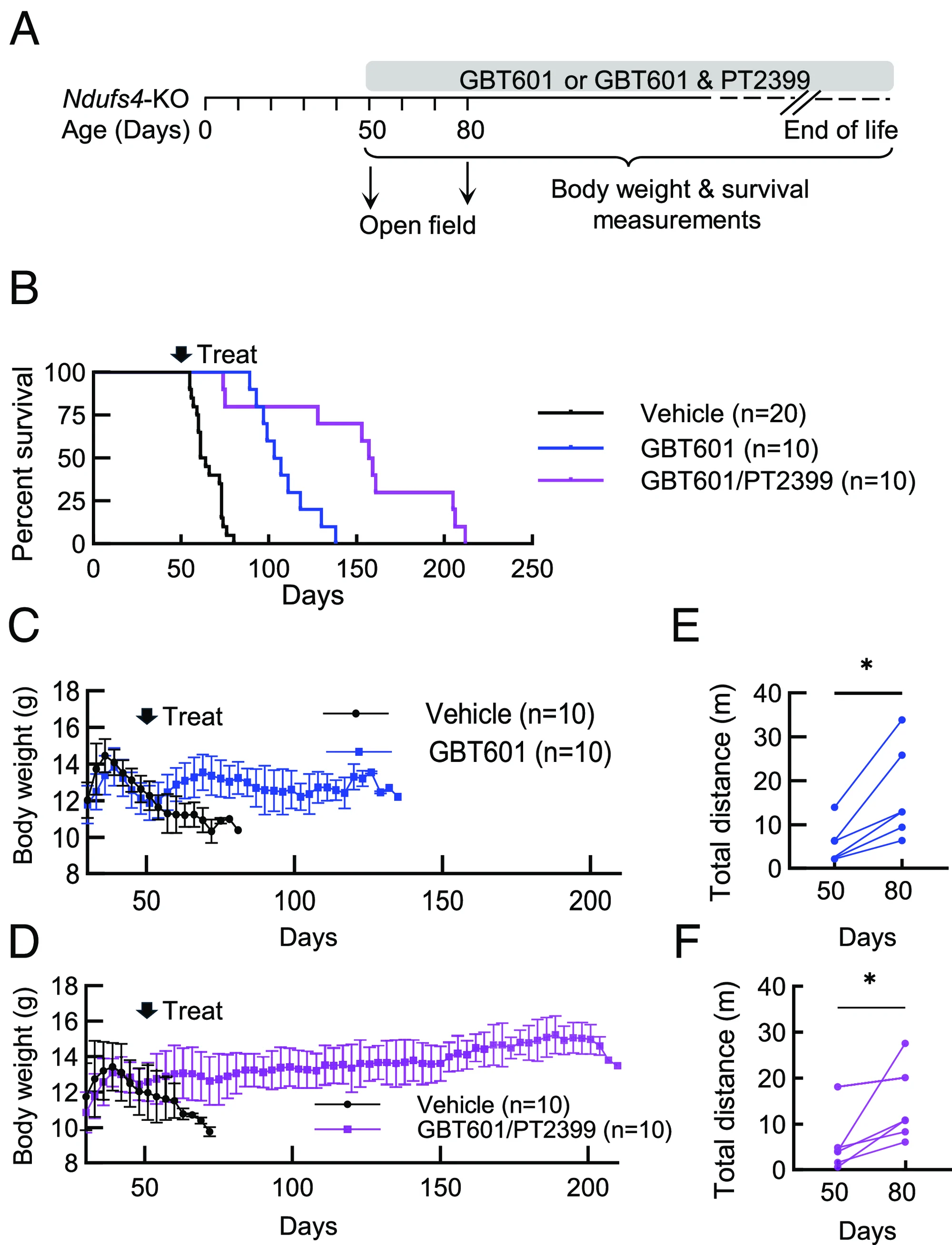
GBT601 alone or GBT601/PT2399 combination alleviates neurological disease and extends lifespan in the *Ndufs4* KO model of Leigh syndrome. (*A*) Schematic depicting the *Ndufs4* KO mice experimental tests and time course. (*B*) Survival of *Ndufs4* KO mice treated with vehicle, GBT601, or the combination. Body weight change of *Ndufs4* KO mice treated with the GBT601 (*C*) or the combination (*D*). Distance traveled in 15 min on an open-field test of *Ndufs4* KO mice treated with GBT601 (*E*) or the GBT601/PT2399 combination (*F*) (n = 6 per group at *E* and *F*). Bar plots show the mean ± SD. *P < 0.05, **P < 0.01, ***P < 0.001, ****P < 0.0001; paired *t* test; log-rank test for survival of drug-vs. vehicle–treated mice. Two-way ANOVA test for body weight of drug-vs. vehicle–treated mice.

Here we sought to evaluate whether the second-generation GBT601 alone, or in combination with PT2399, are effective when initiated at day 50, when these mice exhibit very advanced neurological disease (Fig. 2*A*). This is a more stringent approach (“rescue” vs. “prevention”) and is more clinically relevant as most patients present after the onset of disease symptoms. Ordinarily, *Ndufs4* KO mice are born healthy, show neurological defects on day ~35, begin to lose body weight, and succumb at ~2 mo to their neurodegenerative disease. We began therapy at day 50, when *Ndufs4* KO mice already have advanced neurological disease, and vehicle treated mice are expected to survive for only two more weeks. GBT601 alone increased median lifespan from ~62 d (vehicle) to 105 d (*P* < 0.0001) and maximum lifespan from 80 to 138 d (*P* < 0.0001). Strikingly, the GBT601/PT2399 combination extended median lifespan from ~62 to 158 d (*P* < 0.0001) and maximum lifespan by 2.7 times from 80 to 212 d (*P* < 0.0001, Fig. 2*B*). Moreover, *Ndufs4* KO mice demonstrated an upward growth trajectory following initiation of treatment (Fig. 2 *C* and *D*). At day 70, when half of the vehicle group had died, the average body weight of the GBT601 mice was higher than the surviving vehicle treated mice (13.4 ± 1.1g vs. 10.3 ± 0.6 g, *P* < 0.0001) (Fig. 2*C* and *SI Appendix*, Fig. S1 *A* and *B*). Similarly, the body weight of the GBT601/PT2399 group was higher than the mice surviving in the corresponding vehicle group (12.6 ± 1.4 g vs. 9.8 ± 0.3 g, *P* < 0.0001) (Fig. 2*D* and *SI Appendix*, Fig. S1 *C* and *D*). The open field test showed that distance traveled increased from day 50 prior to treatment to day 80 with either GBT601 (*P* = 0.01, Fig. 2*E*) or with the combination (*P* = 0.048, Fig. 2*F*).

Collectively, these studies indicate that “hypoxia in a pill” monotherapy with GBT601 alone or with the dual-targeting approach GBT601/PT2399 can alleviate neurodegenerative disease phenotypes and rescue the *Ndufs4 KO* mouse when they have advanced disease, with a striking extension of survival and lifespan.

### GBT601/PT2399 Halts Neurological Disease Phenotypes in Friedreich’s Ataxia Mouse Model.

Given the striking performance of the GBT601/PT2399 combination in the *Ndufs4* KO model, we advanced it into a mouse model of Friedreich’s ataxia. Friedreich’s ataxia (FRDA) is the most common inherited ataxia due to a recessive loss of the frataxin (FXN) protein (38–40). Patients are born developmentally intact, and begin to exhibit signs of ataxia ~10 y of age and typically develop diabetes and cardiomyopathy. Using the doxycycline-induced *shFxn* mouse model of Friedreich’s ataxia (41), we previously reported that breathing chronic, continuous 11% oxygen improved ataxia phenotypes (13). We also previously reported that initiating chronic, continuous 11% FIO_2_ for 3 wk can rapidly reverse features of ataxia when commenced 12 wk after doxycycline-induced *shFxn*, at which point neurological debility is advanced (14). However, in contrast to the *Ndufs4* KO mouse model (whose proximal cause of death in normoxia is the brain pathology), hypoxia did not improve lifespan in the *shFxn* model (41), where the cardiac pathology is the presumed proximal cause of death and not alleviated by hypoxia in either model (8, 13).

**Fig. 3.**
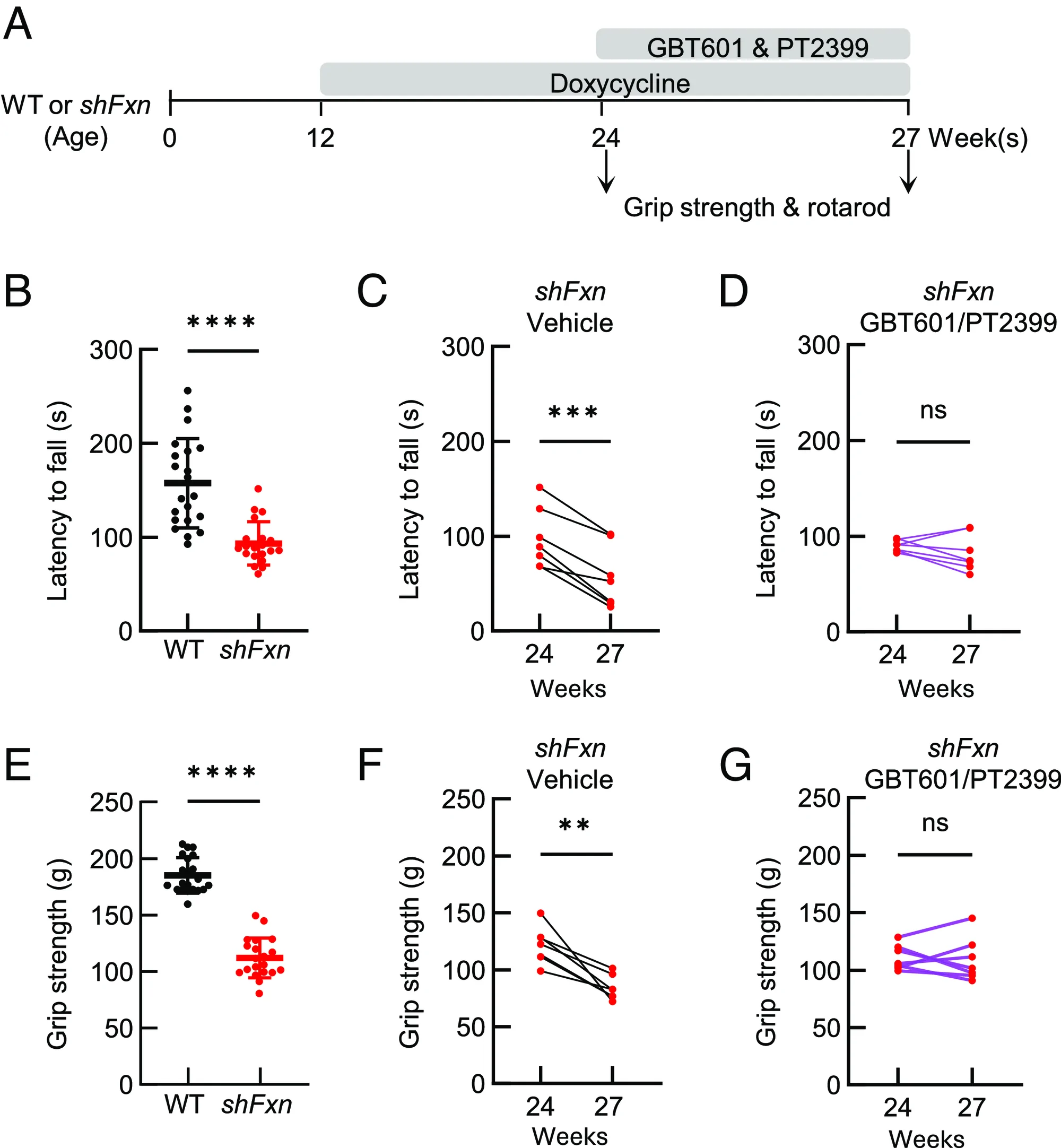
GBT601/PT2399 combination halts further neurological decline in *shFxn* mice. (*A*) Schematic depicting the *shFxn* mice experimental tests and time course. (*B*) Rotarod test in 24-wk-old WT and *shFxn* mice treated with doxycycline for 12 wk (n = 20 or n = 21 per group. Rotarod test in *shFxn* mice treated with vehicle (*C*) or combination (*D*) at 24-wk-old and 27-wk-old. (*E*) Grip strength test in 24-wk-old WT and *shFxn* mice treated with doxycycline for 12 wk (n = 20 or n = 21 per group). Grip strength test in *shFxn* mice treated with vehicle (*F*) or combination (*G*) at 24-wk-old and 27-wk-old (n = 7 per group at *C*, *D*, *F*, and *G*). Bar plots show the mean ± SD. n = group size. *P < 0.05, **P < 0.01, ***P < 0.001, ****P < 0.0001; paired *t* test for single comparison.

Here, we tested whether the GBT601/PT2399 combination could mimic the effects of inhalational hypoxia to similarly halt neurological symptoms in the *shFxn* mice once they began to exhibit signs of advanced debility (Fig. 3*A*). As expected (41), at 24 wk of age (after 12 wk of doxycycline treatment), *shFxn* mice treated with doxycycline began to exhibit decreased latency to fall on rotarod testing (Fig. 3*B*). Between 24 wk and 27 wk, *shFxn* mice treated with the drug vehicle alone continued to exhibit deterioration, with their latency to fall on rotarod testing declining in the surviving mice from a mean of 98 to 57 s (paired *t* test *P* = 0.003, Fig. 3*C*). In contrast, *shFxn* mice treated with GBT601/PT2399 better preserved their time on the rotarod, with a mean latency to fall of 90 s at 24 wk and 83 s at 27 wk in the surviving mice (paired *t* test *P* = 0.3, Fig. 3*D*). At the 24 wk time point, sh*Fxn* mice exhibited marked decrease in grip strength (Fig. 3*E*) and continued to decline from a mean of 122 grams to a mean of 84 grams (paired *t* test, *P* = 0.003, Fig. 3*F*). The GBT601/PT2399 could halt this further decline in surviving mice, with grip strength being preserved from a mean 111 grams at 24 wk vs. mean of 109 grams at 27 wk (paired *t* test, *P* = 0.8, Fig. 3*G*).

Hence, these studies indicate that initiating the combination treatment at 24 wk of age, can halt further progression of the ataxia phenotype, though in contrast to 11% FIO_2_ (13), is unable to reverse these motor phenotypes.

### GBT601/PT2399 Reverses Motor Phenotypes in the α-Syn Preformed Fibrils (PFF)-Induced Mouse Model of PD.

Next we sought to determine whether our “hypoxia in a pill” combination might be effective in a mouse model of PD. PD is the second most common neurodegenerative disease. PD is characterized by a progressive deterioration of both motor and nonmotor symptoms. At a histopathological level, the hallmarks of PD are the loss of dopaminergic (DA) neurons in the substantia nigra and the formation of Lewy bodies, mainly consisting of aggregated fibrils of the α-synuclein (α-syn) protein. Secondary mitochondrial defects, notably in complex I, are frequently documented in sporadic and rare forms of this disease.

We previously reported the therapeutic benefits of breathing chronic, continuous 11% oxygen in a α-syn PFF mouse model of PD (20). In this model, PFFs induce endogenously expressed α-syn to form aggregates closely resembling Lewy Pathology in PD, leading to significant reduction of dopamine neurons in the substantia nigra pars compacta (SNpc) (42). We showed that PFF-induced α-syn aggregation resulted in brain tissue hyperoxia, lipid peroxidation, and dopaminergic neurodegeneration in the SNpc of mice breathing 21% oxygen. In mice breathing 11% oxygen, we found no difference in the abundance of α-syn aggregates, however, hypoxia attenuated the brain hyperoxia, lipid peroxidation, and DA neuron loss. Moreover, initiating hypoxia 6 wk after PFF injection could reverse motor symptoms and halted further DA neurodegeneration (20).

**Fig. 4.**
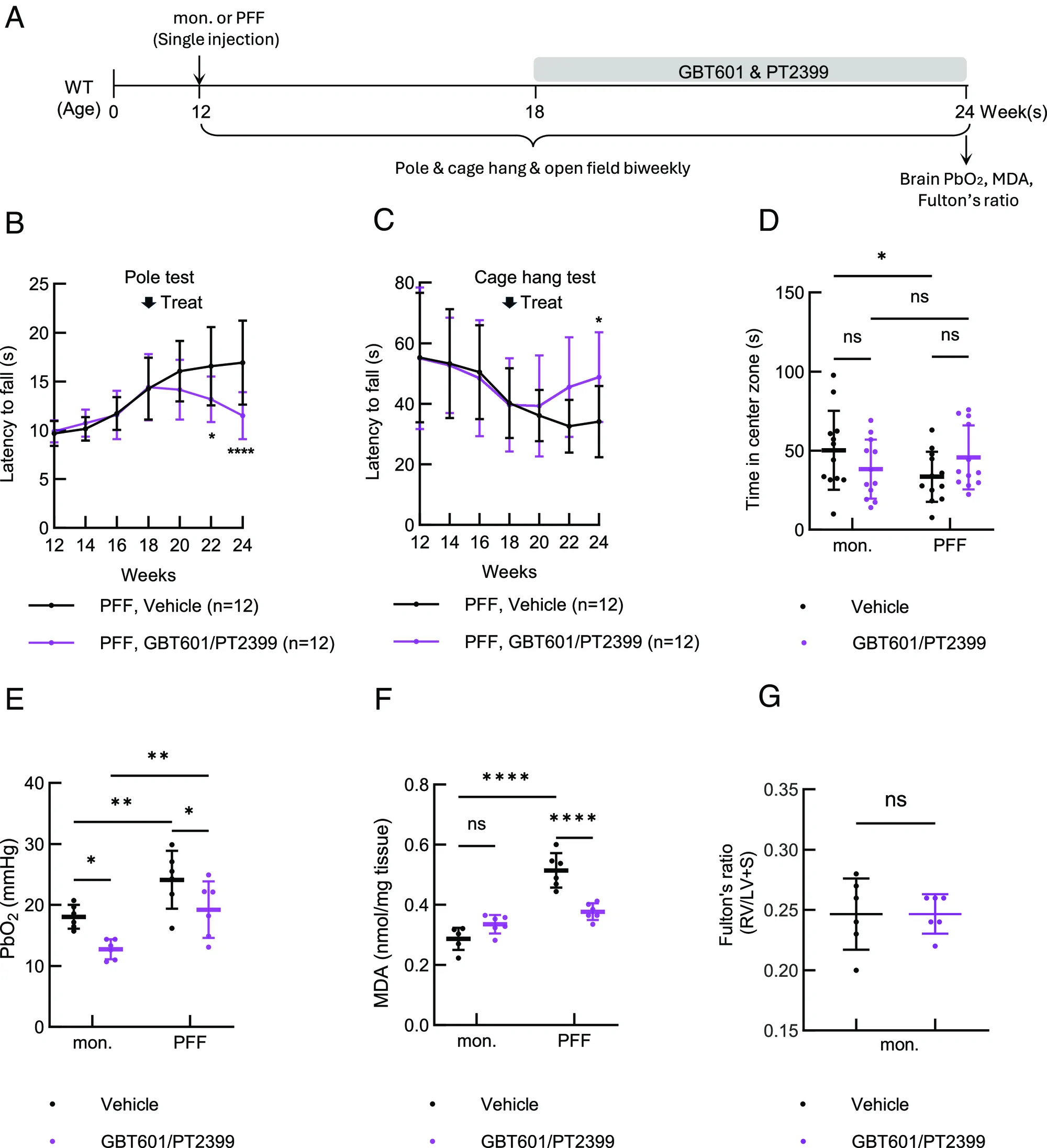
GBT601/PT2399 combination rescues α-syn-induced parkinsonism in mice. (*A*) Schematic depicting the mouse model of PD experimental tests and time course. (*B*) Pole test and (*C*) Cage hang test in PD (PFF) mice treated with vehicle or the GBT601/PT2399 combination. (*D*) time in center zone on the open-field test of control (mon) and PD (PFF) mice treated with vehicle or the GBT601/PT2399 combination. (*E*) brain PbO_2_ measurements (*F*) MDA levels in SNpc tissues of control (mon) and PFF mice treated with vehicle or the GBT601/PT2399 combination. (*G*) Fulton’s ratio of control (mon) mice treated with vehicle or the GBT601/PT2399 combination. Bar plots show the mean ± SD. n = group size, n = 12 per group at *D*. n = 6 per group at *E*–*G*. *P < 0.05, **P < 0.01, ***P < 0.001, ****P < 0.0001. A two-way ANOVA for multiple comparisons was conducted.

We utilized a similar study design but now commenced with the GBT601/PT2399 “hypoxia in a pill” combination (instead of inhalational hypoxia) after disease onset (Fig. 4*A*). Briefly, we administered intrastriatal injections of PFFs or monomeric α-syn (mon) as a control to 12-wk-old mice to confirm onset of motor dysfunction at 18 wk. We then treated the PFF-injected mice or monomer-injected mice with either GBT601/PT2399 combination regimen or vehicle from 18-wk-old to 24-wk-old and assessed motor behavior using the pole test (coordination, bradykinesia), the cage hang test (muscle strength, endurance), and the open field test (gross motor and anxiety-like behavior) (43).

Mice injected with PFFs exhibited increased latency to descend on pole testing from a mean of 9.7 s at 12 wk to 14.3 s at 18 wk, suggesting development of bradykinesia. Between 18 wk and 24 wk, mice treated with the vehicle alone continued to exhibit deterioration, with their latency to descend on pole testing increasing from a mean of 14.3 s (18 wk) to 16.6 s (22 wk) and 16.9 s (24 wk). In contrast, PFF-injected mice treated with GBT601/PT2399 decreased their latency to descend on the pole test, from a mean of 14.4 s (18 wk) to 13.2 s (22 wk) (*P* = 0.02, vs. vehicle) and 11.5 s (24 wk) (*P* < 0.001, vs. vehicle, Fig. 4*B*). Mice injected with PFFs showed marked impairment in the cage hang test. Between 12 wk and 18 wk, latency to fall in the cage hang test decreases from a mean of 55 s at 12 wk to a mean of 40 s at 18 wk. Between 18 wk and 24 wk, mice treated with the vehicle alone continued to exhibit deterioration, with their latency to fall on cage hang test decreasing from a mean of 40 s at 18 wk to 34 s at 24 wk. In contrast, PFF-injected mice treated with GBT601/PT2399 increased their mean latency to fall from 40 s at 18 wk to 49 s at 24 wk (*P* = 0.03, vs. vehicle, Fig. 4*C*). In open field testing, α-syn PFF treated mice showed decreased time in the inner zone (*P* = 0.04) but the drug combination did not improve this phenotype (Fig. 4*D*).

We previously reported that the α-syn PFF model exhibits brain hyperoxia and elevated levels of malondialdehyde (MDA), both of which were attenuated by chronic, continuous hypoxia breathing (20). We sought to determine whether “hypoxia in a pill” is also able to attenuate levels of brain dissolved oxygen and MDA. We measured PbO_2_ in the SNpc 12 wk after intrastriatal injection of PFF or monomer. Mice injected with PFF exhibited higher PbO_2_ (24.1 ± 4.7 mmHg) compared to mice injected with α-syn monomer (18.1 ± 1.9 mmHg, *P* < 0.01) which was attenuated by the combination treatment (19.2 ± 4.6 mmHg, Fig. 4*E*). Mice with α-syn aggregates exhibited higher MDA levels (0.5 ± 0.06 mol/mg) compared to monomer-injected mice (0.3 ± 0.04 mol/mg) (*P* < 0.0001). The “hypoxia in a pill” drug combination attenuated this PFF-induced increase in MDA levels (0.4 ± 0.03 mol/mg, *P* < 0.0001 PFF vehicle vs. PFF GBT601/PT2399, Fig. 4*F*).

Chronic hypoxia breathing is well known to drive pulmonary vascular remodeling in adults, including muscularization of small pulmonary arteries, increased pulmonary vascular resistance, and the development of PH with consequent RV hypertrophy and, in severe cases, RV failure (22, 23). Fulton’s ratio—the weight of the RV free wall divided by the weight of the left ventricle plus septum—is a well-established marker of PH. In mice, exposure to 11% hypoxia for 4 to 8 wk typically increases this ratio from ~0.25 to ~0.35 (24). To determine whether the hypoxia in a pill drug combination therapy induced RV hypertrophy indicative of PH, we measured Fulton’s ratio in monomer-injected mice treated with vehicle or the GBT601/PT2399 combination for 6 wk. Fulton’s ratio was similar in both groups (*P* = 0.2, Fig. 4*G*), suggesting that the drug combination did not induce PH.

## Discussion

In the current paper we have demonstrated that second generation, small molecule based “hypoxia in a pill” regimens are able to rescue disease phenotypes in multiple models of neurodegeneration. We focused on models in which we previously reported that chronic, continuous 11% FIO_2_ was effective. In the *Nudfs4* KO mouse model of Leigh syndrome, we initiated “hypoxia in a pill” therapy at day 50, when mice already have advanced disease and typically survive for less within 2 wk; GBT601 monotherapy extended lifespan from 62 to 105 d of age, while GBT601/PT2399 extended lifespan to 158 d. In the mouse model of Friedreich’s ataxia, initiating dual-targeting therapy at advanced neurological disease helps to halt further deterioration of motor phenotypes. In a model of PD, initiating dual-targeting therapy after onset of advanced disease attenuates brain tissue hyperoxia and MDA levels and reverses motor dysfunction. A compelling aspect of our “hypoxia in a pill” combination is that it appears to be tolerated without any obvious adverse effects on body weight or cardiac remodeling.

The combination appears to perform particularly well in the *Ndufs4* KO mouse model of Leigh syndrome and α-synuclein toxicity model of PD, whereas the effects are more modest in the *shFxn* model of Friedreich’s ataxia. This pattern resembles what we have reported for inhalational hypoxia, too. Chronic continuous hypoxia alleviates neurological disease in the mouse models of Leigh syndrome and Friedreich’s ataxia, however, both of these models also exhibit cardiac disease, and for reasons we do not understand, cardiac disease in these models is not alleviated by hypoxia therapy. As the proximal cause of death in the *Ndufs4* KO model is their brain disease, these mice exhibit a striking lifespan extension with hypoxia therapy or as we have shown now, with the GBT601/PT2399 combination. In contrast, the proximal cause of death in the *shFxn* mouse model appears to be cardiac in origin and their lifespan is not extended by inhalational hypoxia or by “hypoxia in a pill” therapies. The *Ndufs4* KO mouse model and the α-synuclein toxicity model of PD represent models of primary and secondary complex I deficiency, and we have documented the presence of brain hyperoxia (9, 20), which we presume is a consequence of electron transport chain deficiency. We have previously shown that this brain hyperoxia is alleviated by chronic, continuous 11% FIO_2_. Here we have again documented the presence of brain hyperoxia in the PD model, and that it is attenuated by the dual combination therapy. It may be the case that disorders associated with complex I deficiency and/or brain hyperoxia are particularly amenable to systemic hypoxia therapy. Future studies are required to determine the specific cause of death in the *Ndufs4* KO and *shFxn* mouse models treated with the “hypoxia in a pill,” and how they compare to inhalational hypoxia itself.

It is notable that our small molecule-based, dual targeting approach—rationally designed to induce tissue hypoxia while preventing its compensatory erythropoietic response—closely parallels the genetic adaptations that naturally underwent positive selection in human populations living at high altitude. The ascent to high altitude is associated with a compensatory, HIF-2α-dependent erythropoiesis that strives to increase oxygen carrying capacity. This response is initially adaptive, but when prolonged can lead to detrimental side effects. Over evolutionary timescales, in certain populations such as the Tibetans, the chronic environmental pressure of high-altitude hypoxia has favored the selection of specific genetic variants in the EPAS1 (HIF-2α) pathway that blunt its activity (44–46). These adaptations prevent the maladaptive, excessive erythropoiesis typically seen in chronic mountain sickness, thereby protecting the cardiopulmonary system from the complications of hyperviscosity. In mouse models, heterozygous loss of HIF-2α is protective against hypoxia induced PH and right ventricular (RV) dysfunction (27). Our results demonstrate that this genetic mechanism that has been selected for in high altitude adapted human populations can be recapitulated pharmacologically with the HIF-2α inhibitor.

While the current work underscores the promise of a second-generation “hypoxia in a pill,” significant safety and regulatory hurdles remain. Although both drug classes are FDA approved, the first-generation HbO_2_ stabilizer GBT440 (Voxelotor) has been voluntarily withdrawn from the market due to an excess of deaths attributed to vaso-occlusive crises (VOCs) (33). This drug was initially approved on the basis of a biomarker response, and postapproval surveillance revealed continued VOCs on the drug, which is currently under investigation. We note that the physiological consequences of increased hemoglobin oxygen affinity may differ substantially between sickle cell disease—characterized by chronic anemia, microvascular dysfunction, and abnormal hemoglobin polymerization—and a nonsickle context with intact oxygen delivery reserve. Here, we utilized GBT601, a follow-on compound with an improved half-life and higher hemoglobin occupancy that has demonstrated a favorable safety profile in preclinical models and Phase I human trials (34, 35). Nevertheless, elucidating the etiology of class-associated VOCs is essential, and “hypoxia in a pill” regimens may ultimately be contraindicated in patients with sickle cell disease (47). Furthermore, a key caveat remains regarding the use of HIF-2α inhibitors; while FDA-approved for certain renal cancers, they frequently induce anemia, though we leverage this property to counteract the polycythemia typically induced by enhanced hemoglobin affinity. Continued preclinical and eventual clinical investigations of “hypoxia in a pill” regimens are vital to ensuring a responsible path toward confirming their safety, tolerability, and ultimate efficacy in diseases with high unmet medical need.

## Materials and Methods

### Animals.

All animal experimentation was approved by the Massachusetts General Hospital Institutional Animal Care and Use Committee. Mice had free access to food and water and were maintained in a 12-h light–dark cycle, at a temperature between 20 °C and 25 °C and humidity between 40% and 60%. The design of experiments involving animals followed guidelines provided by “Animal Research: Reporting of In Vivo Experiments” guidelines.

We used 10 to 12-wk-old, age-matched and weight-matched male C57BL/6J mice (Jackson Laboratory). To minimize variability, we used a randomized paired (matched pairs) design.

*Ndufs4* KO mice were generously provided by the Palmiter laboratory (University of Washington) (48, 49). As in our prior study (28), pups were genotyped and weaned at 21 to 28 d of age. Natural death or 20% body weight loss (euthanasia criteria) were used to generate survival curves. For the *Ndufs4* KO mouse experiments, data from both sexes are combined as no sex differences in survival phenotypes have been reported.

As in our prior studies (13, 14), *shFxn* mice were kindly provided by the Geschwind Laboratory (UCLA). Pups were genotyped and weaned at ~25 d of age. Animals were balanced by age and sex and entered the study at an average of 3 mo of age. Doxycycline was administered in drinking water (2 mg/mL, replaced weekly) and by intraperitoneal injection twice weekly (5 mg/kg for 10 wk, then 10 mg/kg) (41). Body weight was monitored regularly, and mice were euthanized after a 20% loss of peak body weight.

For PD mouse studies, α-syn synthesis and purification was performed at the University of Alabama at Birmingham (50). As previously described (20), at 3 mo of age, C57BL/6J mice were anesthetized with isoflurane on a stereotaxic frame (MM 8000/3, ASI instruments). Animals were unilaterally injected with 2 μL of 300 μM sonicated fibrils, or 300 μM monomeric α-syn as a control, into the right striatum. Solutions were injected at a constant rate of 0.5 μL min^−1^ using a gastight syringe (catalog #80030, Hamilton, Reno, NV) and a microinjection syringe pump (World Precision Instruments, Sarasota, FL) and the needle was left in place for 2 min; this was followed by slowly withdrawing the needle. The coordinates for the right striatum were +0.2 mm anteroposterior (AP), ±2.0 mm mediolateral (ML) and −3.4 mm dorsoventral (DV). All mice survived after stereotaxic injection of α-syn monomer or PFF and were used for the following experiment.

### Drug Administration.

We purchased GBT601 and PT2399 from MedChemExpress and used dosing regimens as previously described (28, 36). Mice were treated via oral gavage daily at a dose of 100 mg/kg of PT2399 and 150 mg/kg of GBT601. Vehicle-treatment refers to either the vehicle for PT2399 (10% ethanol, 0.5% methyl cellulose/phosphate buffered saline) or the vehicle for GBT601 (10% DMSO, 0.5% methyl cellulose/phosphate buffered saline).

### Hemoglobin Measurements.

As we reported previously (14, 28), blood samples (80 μL) were obtained by tail snip and collected into heparinized capillary tubes. Hemoglobin concentration was measured using an ABL800 FLEX blood gas analyzer (Radiometer, Copenhagen, Denmark).

### Brain Tissue PO_2_ Measurement.

Brain tissue PO_2_ was measured as previously (20, 28). For PbO_2_ measurements in the vestibular nucleus, mice were anesthetized using isoflurane (induction at 2 to 4%, maintenance at 1.5%), intubated, and ventilated mechanically using a tidal volume of 8 mL/kg, a respiratory rate of 110 breaths per minute, and FIO_2_ of 21%. For PbO_2_ measurements in the SNpc, mice were anesthetized with isoflurane (induction at 2 to 4%, maintenance at 1.5%) with FIO_2_ of 21% via a nose cone. Mice were placed in a prone position and the head stabilized using a stereotaxic frame (ASI Instruments, MI). Following skin incision and dissection, the skull was opened using a microdrill (MD-1200, Braintree Scientific, MA) and a PO_2_ probe was inserted at the desired location. The coordinates were ML = −1.25 mm, AP = −6.00 mm, and DV = −3.90 mm or ML = 1.20 mm, AP = −3.20 mm and DV = −4.40 from the bregma for vestibular nucleus or SNpc, respectively. We measured brain tissue PO_2_ using an optical PO_2_ probe (OxyLab, Oxford Optronix, Abingdon, UK). During these brain PO_2_ measurements, we reduced the depth of anesthesia by decreasing Isoflurane concentration to 1% to minimize the effects of anesthesia.

### Oxygen Dissociation Curve Determination.

Twelve-week-old mice received daily oral gavage with vehicle, PT2399, GBT601, or the combination of PT2399 and GBT601 for seven consecutive days (n = 4 to 5 mice per group). Two to three hours after the last administration, mice were anesthetized with isoflurane, underwent tracheostomy, and were mechanically ventilated. The right carotid artery and right jugular vein were cannulated with PE-10 catheters for arterial and venous blood sampling, respectively. To generate oxygen dissociation curves, inspired oxygen fraction (FiO_2_) was stepwise adjusted to achieve a broad range of arterial and venous oxygen tensions (PaO_2_). At each FiO_2_ level, arterial and/or venous blood gases were obtained. Blood samples were analyzed immediately using a blood gas analyzer (ABL90 FLEX PLUS Radiometer). Oxygen saturation values used for curve construction were calculated as functional hemoglobin oxygen saturation (SO_2_) to account only for hemoglobin species capable of binding oxygen. Functional saturation was calculated as

$$SO_{2}\;=\;\frac{O_{2}Hb}{O_{2}Hb\;+\;HHb},$$

where HHb represents deoxyhemoglobin and is calculated as 100 − O_2_Hb − COHb − MetHb. Oxygen dissociation curves were constructed by plotting functional SO_2_ against corresponding PaO_2_ values. Data were analyzed using GraphPad Prism. Curves were fitted by nonlinear regression using a four-parameter logistic equation (log[agonist] vs. response). The upper and lower plateaus were constrained to 100% and 0% saturation, respectively, while P50 and Hill slope were allowed to vary freely.

### Accelerating Rotarod Measurements.

We performed rotarod measurements as previously reported (13, 14), using a device available from Ugo Basile. Mice were acclimated to the testing room for 30 min prior to assessment. The rod accelerated at 5 rpm/min to a maximum speed of 40 rpm. Each testing session consisted of three trials separated by a minimum 10-min rest period, and the median latency to fall was recorded. If mice used their body to grasp the rod (rather than walking on it) for more than 10 s, this time was recorded as time of fall.

### Grip Strength Test.

Forelimb and hindlimb grip strength were measured using a grip strength meter (Bioseb). Each mouse completed three consecutive efforts with 30 s of rest in between measurements. The highest force generated (g) was recorded. If the time period of gripping was less than 10 s, that experiment was not included. Each mouse was tested three times, and the trials were averaged.

### Open Field Test.

We performed open field testing as previously reported (28), The open field apparatus consisted of four open-field boxes measuring 50 × 50 cm. Each box was surrounded by opaque black walls, and the whole apparatus was shielded from outside influences by walls with a height of 1.5 m. After an acclimation period of 60 min inside the procedure room, one mouse was placed in the center of an open-field box. Up to four mice were tested in the apparatus at once. The animals were allowed to explore the field freely for 15 min while being recorded by the ANYaze software with an overhead camera (v7.3, Stoelting Co., Wood Dale, IL). The position and movement of the animals were automatically tracked and analyzed by the ANY-maze software. The experimenters were blind to the treatment groups and, after starting the recording, left the room for the duration of the test. Before and after each test run, 70% ethanol was used to clean the apparatus.

### Pole Test and Cage Hang Test.

We performed behavioral tests as previously reported (20). Behavioral tests were conducted between 8:00 and 12:00 for the pole test and the cage hang test or 12:00 and 17:00 for the open field test. To conduct the pole test, a vertical metal pole with 2-cm diameter and 40-cm length was placed on the floor of a housing cage. Mice were placed on the top of the vertical metal pole and the time needed to reach the floor was measured. To conduct the cage hang test, a metal wire frame of the housing cage (WBL7115SMD-AMG, Allentown) was placed approximately 40 cm above the cage floor. Mice were hung on the metal wire frame and the time needed to drop onto the cage floor was measured. Each test was conducted five times and the average of the last three trials was used for the analysis.

### Detection of MDA.

We measured the concentration of MDA in the SN tissue to evaluate the effect of drugs using a commercially available kit (cat. no. ab118970, Abcam). The assay was conducted according to the manufacturer’s protocol.

### Fulton’s Ratio Measurement.

Mouse hearts were excised from the thoracic cavities, the atria removed, and the free wall of the right ventricle was separated from the left ventricle and the septum. The ratio of the weight of the right ventricle to the weight of the left ventricle and the septum was calculated (Fulton’s ratio). All measurements were performed by an investigator blinded to the treatment groups.

### Quantification and Statistical Analysis.

Data are reported as mean ± SD. Statistical analyses were performed using GraphPad Prism 10.6.1 software. The two-way ANOVA test was used for multiple comparisons, one-way ANOVA with Dunnett’s test for multiple comparisons with vehicle, and the paired *t* test for single comparisons for drugs to vehicle. *P*-value < 0.05 was considered to indicate statistical significance. A log-rank test for survival of drug-vs. vehicle–treated mice. oxygen–hemoglobin dissociation curve compared by nonlinear regression (extra sum-of-squares F test).

## Supplementary Material

Appendix 01 (PDF)

## Data Availability

Study data are included in the article and/or *SI Appendix*.
