# Supplementary material for: A second-generation “hypoxia in a pill” rescues neurodegenerative phenotypes across distinct mouse models
Source: Proc Natl Acad Sci U S A. 2026 Oct 9;123(41):e2618654123. doi: 10.1073/pnas.2618654123 (PMC13663714; doi:10.1073/pnas.2618654123)
Supplement: Supplementary file 1 [file pnas.2618654123.sapp.pdf]

PNAS

Supporting Information for

A second-generation "hypoxia in a pill" rescues neurodegenerative phenotypes across distinct mouse models

Hong Wang[1,2,3], Eizo Marutani[4], Luca Zazzeron[4], Marissa Menard[5], Laura Volpicelli-Daley[5], Fumito Ichinose[4], Vamsi K. Mootha[1,2,3]

1. HHMI and Department of Molecular Biology, Massachusetts General Hospital, Boston, MA 02114
2. Department of Systems Biology, Harvard Medical School, Boston 02115
3. Broad Institute of Massachusetts Institute of Technology and Harvard, Cambridge, MA 02142
4. Anesthesia Center for Critical Care Research of the Department of Anesthesia, Critical Care and Pain Medicine, Massachusetts General Hospital and Harvard Medical School, Boston, MA 02114
5. Killion Center for Neurodegeneration and Experimental Therapeutics, Department of Neurology, University of Alabama at Birmingham, Birmingham, AL 35294

*Corresponding author: Vamsi K. Mootha, M.D.
Email: vamsi@hms.harvard.edu

This PDF file includes:

Figure S1

Fig. S1.

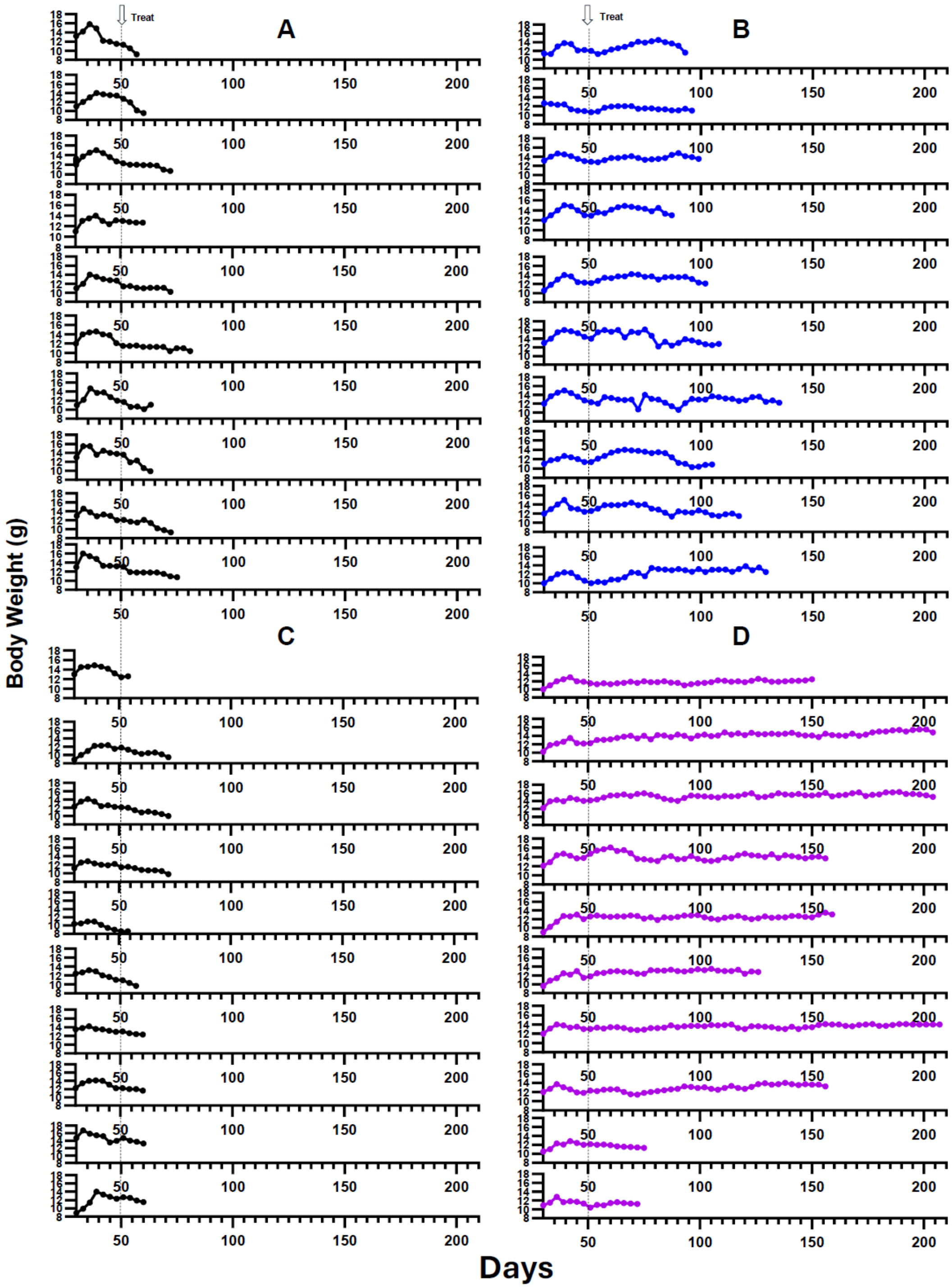

Fig. S1. Effects of "hypoxia in a pill" regimens on growth trajectory of *Ndufs4* KO mice.
Individual mouse body weight trajectories for *Ndufs4* KO mice treated with (**A**) vehicle, (**B**) GBT601, (**C**) vehicle, or (**D**) GBT601/PT2399.
